# Parenteral nutrition: a life–saving intervention for 4 months in short bowel syndrome—a case report and review of the literature

**DOI:** 10.1186/s13256-024-04442-1

**Published:** 2024-03-21

**Authors:** Saulius Švagždys, Ieva Smolskaitė, Rūta Vindžigalskytė

**Affiliations:** 1 Department of Abdominal Surgery, Lithuanian University of Health Sciences, Kaunas, Lithuania; 2 Faculty of Medicine, Lithuanian University of Health Sciences, Kaunas, Lithuania

**Keywords:** Bowel resection, Short bowel syndrome, Malabsorption, Parenteral nutrition, Case report

## Abstract

**Background:**

Short bowel syndrome (SBS) in adults is defined as having less than 180 to 200 cm of remaining small bowel. Many literature sources do not provide precise epidemiological data, and challenges in estimating the prevalence of SBS include its multifactorial etiology and varying definitions. The most common pathologies leading to SBS include Crohn disease, mesenteric ischemia, radiation enteritis, post-surgical adhesions, and post*-*operative complications.

**Case presentation:**

This article presents a clinical case of a 76-year-old Lithuanian patient who underwent parenteral nutrition for four months due to SBS. Before the following diagnosis, the patient had undergone two surgeries. During the hospitalization, life-threatening conditions such as stercoral peritonitis, septic shock, and acute respiratory failure, were observed and treated. As a result of SBS, hypoproteinemia and hypoalbuminemia developed, leading to the prescription of full parenteral nutrition. After correcting the malnutrition, a third surgery was performed, resulting in the discontinuation of parenteral nutrition and the resumption of a regular diet.

**Conclusions:**

Parenteral nutrition is the sole effective method for preserving the lives of patients with a short segment of the intestine. While on parenteral nutrition, patients can be prepared for reconstructive surgery.

## Background

Short bowel syndrome (SBS) in adults is defined as less than 180 to 200 cm of remaining small bowel, whereas the normal range extends from 275 to 850 cm [1–4]. The prevalence of SBS in the general population is approximately 1–2 cases per 100,000 inhabitants per year. In the USA, the incidence ranges from 0.3 to 4 cases per 100,000 inhabitants annually, while in Europe, it varies from 0.1 to 4 cases per 100,000 inhabitants per year [5]. The lack of precise epidemiological data in numerous literature sources poses challenges in estimating the prevalence of SBS, attributed to its multifactorial etiology and varying definitions [1–7]. The most common pathologies leading to SBS include Crohn disease, mesenteric ischemia, radiation enteritis, post-surgical adhesions, and post*-*operative complications [1, 2, 4–6]. Intestinal resection surgery reduces nutrient absorption, alters motility and microbiota, and leads to malabsorption. Clinical manifestations include weight loss, diarrhea, steatorrhea, dehydration, nutritional deficiency, and electrolyte imbalance [1–4, 6].

In severe cases of SBS, parenteral nutrition (PN) might become necessary to administer essential nutrients directly into the bloodstream, circumventing the gastrointestinal tract (GIT) [1, 8–10]. Alternatively, nutrition can be provided enterally by delivering mixtures into the GIT using an enteral tube or stoma, or through a combination of these artificial feeding methods [11, 12]. Enteral nutrition (EN) is the preferred method due to its lower complication rates and cost-effectiveness. However, in situations where enteral feeding is contraindicated, or GIT dysfunction is present, full parenteral nutrition (FPN) becomes essential. The main indications for PN and EN are listed in Table 1. EN is commonly indicated for neurological diseases and cancer, while PN is recommended for cases of SBS, malabsorption, and mechanical obstruction of the GIT [13, 14].

**Table 1:** Indications for parenteral and enteral nutrition

Indications for parenteral nutrition	Indications for enteral nutrition	
• Short bowel • Chronic intestinal obstruction because of GIT^1^ cancer • Intestinal pseudo-obstruction with food intolerance • Gastrointestinal fistula • Bowel obstruction • Intestinal dysmotility • Prolonged bowel rest • Severe malnutrition, significant weight loss and/or hypoproteinemia when enteral therapy is not possible • Other disease states or conditions in which oral or enteral feeding are not an option	• Neurologic diseases with dysphagia: stroke, motor neuron disease, cerebral palsy, Parkinson’s disease, head trauma • Malignant obstruction: head and neck cancer, esophageal cancer • Benign esophageal stricture • Acute diseases with hypermetabolism: critically ill patients, severe burns, severe acute pancreatitis, cirrhosis • Chronic diseases with hypermetabolism: oncological diseases, chronic lung diseases • Anorexia nervosa, limited oral feeding owing to psychiatric conditions	

^1^Gastrointestinal tract

EN requires the presence of an enteral tube or stoma. Percutaneous endoscopic methods of enteral feeding include gastrostomy, jejunostomy, and gastrojejunostomy. The choice of approach depends on the underlying disease, the patient's nutritional tolerance, and the anatomy of the GIT. In cases where an endoscopic procedure is not possible, radiological or surgical methods are applied. For long-term PN, three types of venous access are employed: a peripheral vein central catheter, a tunneled central vein catheter, and an implantable Ports catheter. These catheters are positioned to terminate either in the superior vena cava or the right atrium [13, 14].

The clinical case in this article emphasizes the importance of PN in ensuring the acquisition of all essential micro and macronutrients in critically ill patients with SBS.

## Case presentation

A 76-year-old Lithuanian man with symptoms of abdominal pain, nausea, and vomiting was urgently admitted to the Department of Esophageal, Gastric, and Endocrine Surgery, on the 1st of October 2022.

The patient has a medical history of type 2 diabetes mellitus, glaucoma, arterial hypertension, chronic atrial fibrillation, benign prostatic hyperplasia, gout, heart failure, bronchial asthma, and ischemic heart disease.

Upon examination, the abdomen revealed scarring from previous surgeries in 2010 for acute diverticulitis and in 2012 for colostomy closure, with tenderness near the umbilicus and on the right side, without other significant findings. Abdominal and pelvic computed tomography (CT) scans indicated signs of small bowel obstruction. Initially, conservative treatment with a nasogastric tube was administered. However, due to the lack of a positive response, the patient underwent urgent surgery, which included laparotomy, release of the small bowel loop, adhesiolysis with bowel wall suture of strangled site, and Douglas cavity drainage. The post-operative diagnosis was mechanical ileus.

Due to postoperative peritonitis-induced disruption of homeostasis and respiratory function insufficiency (RFI), with a persistent requirement for mechanical ventilation (MV), the patient was transferred to the Central Reanimation Unit (CRU). In the CRU, correction of electrolyte imbalance, continuation of infusion therapy, and empirical antibiotic treatment with Cefuroxime and Metronidazole were maintained since the day of surgery, along with prophylaxis of thromboembolism and stress gastric ulcer. Following stabilization of the patient's condition, observation of positive changes in inflammatory markers and absence of clinical signs of peritonitis, the patient was subsequently transferred to the Colon and Perineal Surgery Unit (CPSU) for further treatment.

**Fig. 1:**
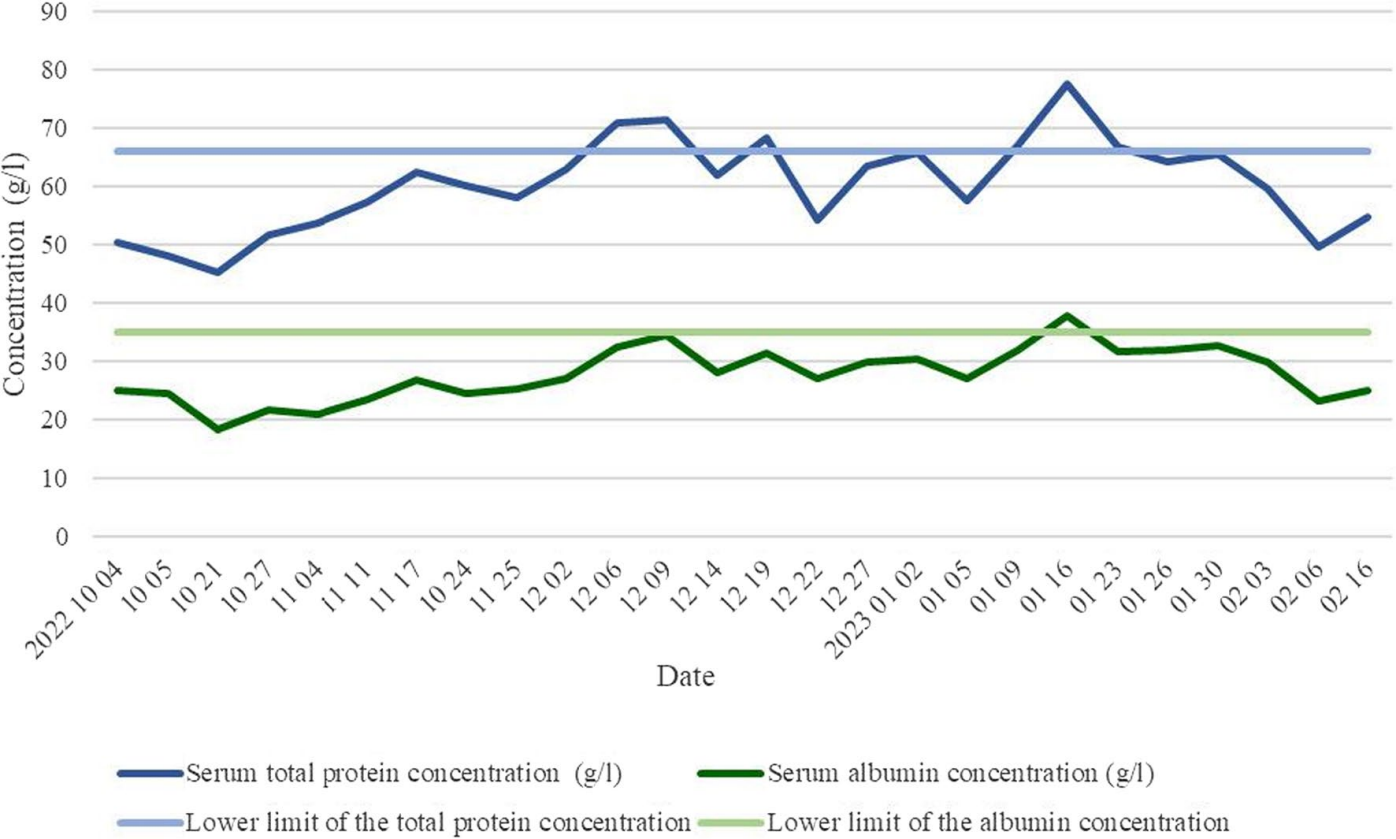
Dynamics of serum total protein and albumin concentrations at the hospital of Lithuanian University of Health Sciences, Kaunas Clinics, from October 1, 2022, to February 16, 2023

Laboratory blood tests were conducted to evaluate the patient's nutritional status, monitoring hypoproteinemia of 50.3 g/l, and hypoalbuminemia of 24.5 g/l (Fig. 1). As part of the treatment plan, a semi-pureed food (SPF) diet was prescribed, supplemented with two bottles of the specialized mix Diasip *(manufactured and registered by N. V. Nutricia, Netherlands)* and 500 ml of 10 % Aminoven *(manufactured and registered by Fresenius Kabi AB, Sweden)* administered intravenously daily.

Five days after the first surgery, the patient developed a fever up to 40 °C, and abdominal pain emerged. Following an abdominal X-ray examination, a positive dynamics of bowel obstruction was observed. As the mentioned symptoms progressed, an abdominal and pelvic CT scan revealed stercoral peritonitis. The patient underwent an urgent relaparotomy, during which a conglomerate of the small intestine with a perforated strangulated loop, located 20 cm proximal to the perforation, was removed, leaving only 70 cm of remaining small bowel. Peritoneal lavage and drainage were performed, and an end-ileostomy was created. A preliminary diagnosis of short bowel syndrome (SBS) was established.

Due to postoperative RFI, sepsis and septic shock, the patient was transferred to CRU. Noradrenaline was administered for perfusion support, along with MV, infusion therapy, prophylaxis for stress ulcers and thromboembolism, correction of electrolyte imbalance, and empirical antibiotic treatment with Meropenem and Vancomycin. Following a gradual reduction in vasoconstrictor support and stabilization of hemodynamics, the patient was transferred to the CPSU, maintaining an ongoing oxygen therapy requirement of 6 l/min. As the treatment progressed, the patient's condition improved, and abdominal ultrasound revealed no signs of intestinal eventration.

Approximately 1 week after the second surgery, with SBS and serum total protein levels at 45.3 g/l and albumin at 18.3 g/l, falling below the lower limit of normal (Fig. 1), the patient received nutritional support. Following catheterization of the right internal jugular vein, full parenteral nutrition (FPN) was initiated, involving SmofKabiven Central *(manufactured and registered by Fresenius Kabi AB, Sweden)* infusion emulsion, as well as micronutrients and vitamin concentrates of Soluvit *(manufactured and registered and produced Fresenius Kabi, France)*, Addaven, and Vitalipid *(both manufactured and registered by Fresenius Kabi AB, Sweden)*. To maintain the trophicity of intestinal villi, the patient continued with an oral SPF diet. For further enhancement of the nutritional status, the patient was admitted to the Nutritional Unit for symptomatic treatment and FPN from December 5, 2022, until January 31, 2023, in preparation for the upcoming restorative intestinal surgery.

In a stable condition, following the correction of hypoproteinemia (increased from 63.0 g/l to 65.5 g/l) and hypoalbuminemia (elevated from 27.0 g/l to 32.8 g/l) (Fig. 1), a laparotomy was performed to close the ileostomy. FPN was continued for 2 weeks postoperatively, extending the total duration to 4 months.

After the restoration of intestinal integrity and a return to a normal diet, the patient’s parenteral nutrition was discontinued, and his nutritional status was corrected (total protein 54.8 g/l and albumin 25.1 g/l) (Fig. 1). Subsequently, the patient was discharged home for further outpatient treatment under the care of a family doctor.

## Discussion

Parenteral nutrition (PN) encompasses a blend of solutions containing dextrose, amino acids, electrolytes, vitamins, minerals, and trace elements. However, the precise formulation and rate of administration are individually tailored [15]. PN is indicated when oral or enteral feeding proves insufficient to address nutritional requirements, or when contraindications to these modalities exist, such as hemodynamic instability, intestinal obstruction, severe vomiting or diarrhea, gastrointestinal hemorrhage, or intestinal ischemia [16].

In cases of short bowel syndrome (SBS), the residual length of the intestine is measured from the duodenojejunal flexure to either the ileocecal junction, the site of any small bowel–colon anastomosis, or the location of the end-ostomy [15]. SBS patients can be classified into three groups based on the presence or absence of residual colon: Group 1, with end-jejunostomy; Group 2, with the jejunum anastomosed to partial colon (jejuno-colic anastomosis); and Group 3, with jejuno-ileo-colic anastomosis, retaining the entire colon and ileocecal valve. It is essential to emphasize that the third group demonstrates the most favorable prognosis for survival, while the first group presents the least favorable outcome, encompassing patients with the most severe condition [17, 18]. In the clinical case presented, the patient underwent an end-ileostomy as a temporary measure preceding restorative surgery.

Following intestinal resection surgery, the gastrointestinal adaptation process commences and can categorized into three phases based on both duration and physiological characteristics. Phase 1, lasting 1-3 months, is marked by the potential onset of severe diarrhea and reduced intestinal absorption. During this period, PN is administered to address nutritional and fluid requirements, preventing the risk of intestinal failure, nitrogen imbalance, and sudden, substantial weight loss. As the gastrointestinal tract (GIT) becomes capable of absorbing food, enteral nutrition (EN) is initiated in the early post-operative phase. This approach stimulates intestinal adaptation through three mechanisms: mucosal hyperplasia, the secretion of gastrointestinal hormones, and the release of pancreaticobiliary system juices and enzymes. Phase 2, lasting up to 1 month, encompasses the influence of remaining intestinal hormones and growth factors that foster functional and structural changes in the GIT. This adaptation facilitates the recovery of the remaining intestinal tract, enhancing absorption by reducing fluid loss and improving the uptake of micro and macro elements. As a result, the use of PN is gradually reduced to increase reliance on EN. In Phase 3, which may last up to two years, the intestinal adaptation reaches its maximum. During this period, PN is either stopped or minimized because the GIT has reached its peak adaptive state [17].

Patients receiving a combination of PN and EN have better treatment outcomes compared to those receiving PN alone. One of the pivotal factors contributing to this observation is the diminished risk of complications, including infections and adverse metabolic reactions such as hyperglycemia, serum electrolyte imbalances, and excessive or insufficient levels of essential nutrients [15]. This clinical case illustrates that despite an adequate intake of micro and macronutrients, suboptimal nutritional status may still occur during treatment. Despite the administration of PN in combination with oral intake, it seems that the supplied food was inadequate, as evidenced by the patient's 15 kg weight loss over the 4-month treatment period. This occurred in the context of nutritional deficiency, prior treatments, and heightened nutritional demands during the postoperative phase, with the patient’s weight decreasing from 110 kg on October 6, 2022, to 95 kg on February 6, 2023. Nevertheless, this weight change did not significantly impact the patient’s overall condition and outcome. This clinical case highlights that partial oral nutrition promotes the growth of intestinal goblet cells, while PN ensures the patient's intake of vital nutrients, and both approaches can effectively complement each other.

The primary goals for patients with SBS involve restoring intestinal integrity and enhancing the function of the remaining intestine through specialized lengthening or narrowing surgeries, aiming to reduce reliance on PN. Each bowel restorative surgery is individually customized [18]. Whenever feasible, the restoration of intestinal continuity, such as re-anastomosis of the small intestine with the colon, should be performed [19, 20]. In patients with SBS complicated by intestinal insufficiency, autologous gastrointestinal reconstructive surgery is undertaken, with the choice of method determined by the existing bowel length, function, and caliber. As SBS progresses, bowel segments undergo expansion to compensate for the reduced surface area and length. When peristalsis slows down and intestinal segments dilate, surgical interventions aim to reduce the intestinal radius while preserving the current absorptive surface area. These procedures encompass longitudinal intestinal lengthening and tapering (LILT) following the Bianchi technique or serial transverse enteroplasty (STEP). For patients with rapid peristalsis but no dilation, surgical interventions such as segmental reversal of the small bowel (SRSB) or isoperistaltic colonic interposition are performed to slow down the evacuation of bowel contents [19, 20].

In this clinical situation, the patient underwent the removal of an end-ileostomy and a side-to-side entero-enteric anastomosis.

## Conclusions

Parenteral nutrition is the sole effective method for preserving the lives of patients with a short segment of the intestine. While on parenteral nutrition, patients can be prepared for reconstructive surgery.

## Data Availability

Data is contained within the article.
